# Macro–Meso Damage Analysis of Tunnel Lining Concrete under Thermal–Mechanical Coupling Based on CT Images

**DOI:** 10.3390/ma17010253

**Published:** 2024-01-03

**Authors:** Xudong Zheng, Wei Wang, Yanfei Zhang, Jinhui Qi, Xuedan Yao

**Affiliations:** School of Civil Engineering, Central South University, Changsha 410075, China; zhengxd@csu.edu.cn (X.Z.); csuqijinhui@csu.edu.cn (J.Q.);

**Keywords:** tunnel lining concrete, fire, CT, mesoscopic deterioration characteristics, macro–meso damage connection

## Abstract

The mechanical properties and failure modes of concrete are controlled by its mesoscopic material composition and structure; therefore, it is necessary to study the deterioration characteristics of tunnel lining concrete under fire from a mesoscopic perspective. However, previous studies mostly analyzed the damage and failure process from a macro-homogeneous perspective, which has certain limitations. In this paper, a thermal–mechanical coupling test device was modified to simulate the state of concrete under tunnel fire conditions. Combined with CT technology, the macroscopic properties and mesoscopic characteristics of concrete were observed. Features were obtained, such as the change in compressive strength under fire, as well as mesoscopic deterioration characteristics. The damage variable D was defined to quantify mesoscopic damage, and the link between mesoscopic deterioration characteristics and macroscopic performance was established, which can be used to predict compressive strength loss through mesoscopic characteristics.

## 1. Introduction

China’s pace in developing transportation infrastructure is remarkable. As evidence, by the close of 2022, the country boasted 24,850 road tunnels with a total length of 26,784 km [1] and 17,873 railway tunnels covering 21,978 km [2]. While these achievements have undeniably brought tremendous convenience, they also come with challenges: the increasing tunnel fire incidents have significantly jeopardized both user safety and the integrity of tunnel structures. Thus, gaining a deep understanding of the failure mechanisms of tunnel linings under high temperatures becomes imperative, especially since these linings are the first line of defense against fire.

Regarding macroscopic damage, numerous studies have delved into the effects of disasters—such as leakage and fire—on tunnel lining concrete. Research has covered aspects such as the stress of concrete, the duration of fire exposure, and the resulting deformations under varying fire intensities [3,4,5]. Guo et al. [6] investigated the correlation between internal damage and applied loads in high-temperature-affected concrete. Maluk et al. [7] conducted experimental investigations to study the fire behavior and integrity of macro polypropylene fibers in fiber-reinforced concrete after fire exposure. Other notable studies encompassed post-fire residual compressive strengths of high-performance concrete [8], relationships between temperature and the accompanying color change in concrete [9], and the temporal variation in concrete temperature and stress during a fire [10]. Some research works conducted large-scale model experiments [11] or employed real-world tunnel scenarios [12] to assess the post-combustion mechanical properties of concrete linings. Moreover, a fire test was carried out on shield tunnel linings in order to evaluate the condition of concrete through residual strength and spalling behavior [13].

With regard to concrete mesoscopic damage, the application of computed tomography (CT) technology in assessing damage in concrete has seen a notable increase. This technique provides a quantitative insight into the internal damage of concrete samples, shedding light on both the mechanics and the evolution of mesoscopic damage. There has been research focusing on the microstructural damages to concrete under static loads [14] or dynamic loads [15]; another study analyzed mesoscopic characteristics of concrete samples drilled from a prefabricated tunnel lining element after a fire [16]; and additional research probed into tunnel lining damage distribution under the heat impact coupling effect using CT scanning [17]. There has also been research utilizing X-ray CT images to reveal the importance of microstructure recovery in restoring the performance of concrete damaged by fire [18].

However, there are two gaps in previous studies. First, many studies perceive tunnel lining concrete as a homogeneous macro entity, neglecting the effects of heterogeneity of the internal medium. Second, while numerous CT-based tests concern meso damage induced by either load or temperature, few consider their combined effects. Thus, there is a clear paucity of insights on the mesoscopic degradation features of tunnel lining under fire. Given that concrete’s mechanical properties and failure mechanisms are largely influenced by its mesoscopic composition and structure [19], bridging the knowledge gap between macro and meso attributes is crucial. Moreover, previous tests did not adopt the single-side fire condition, a scenario more akin to the in-service lining concrete under actual fire incidents. Therefore, there is room for further improvement in the test condition.

In this study, an experimental setup simulating the actual fire exposure conditions of lining concrete is improvised by integrating a thermal–mechanical coupling device. The research investigates the influence of two variables—the duration of fire exposure and the coarse aggregate content—on the macroscopic mechanical properties of lining concrete. By employing industrial CT scanning, the study observes the mesoscopic degradation characteristics of lining concrete under various conditions and quantitatively characterizes the extent of mesoscopic damage. Ultimately, the study seeks to explore the relationship between changes in macroscopic mechanical properties and mesoscopic damage, with the objective of revealing the connection between the macroscopic and mesoscopic aspects of lining concrete.

## 2. Materials and Methods

### 2.1. Concrete

The concrete specimens utilized in this study are of C50 grade, mirroring the mix employed in the construction of the Hong Kong-Zhuhai-Macao immersed tunnel. The materials include P.O 42.5 ordinary Portland cement, density of 3140 kg/m^3^; coarse aggregate, which is a limestone crushed stone (5–10 mm small gravel and 10–20 mm big gravel), density of 2680 kg/m^3^; sand with fineness modulus 2.6, zone 2, good gradation, density 2600 kg/m^3^; F grade fly ash, density 2600 kg/m^3^; S95 grade slag, density 2900 kg/m^3^; polycarboxylate superplasticizer; tap water. The concrete mix ratio is shown in Table 1.

### 2.2. Sample Preparation

Referring to the Standard for Test Methods of Concrete Physical and Mechanical Properties (GB/T50081-2019) [20], each concrete cube has a side length of 100 mm. The prepared specimens are placed in an environment of 20 ± 5 °C for 24 h prior to demolding. After demolding, the samples are immersed in a saturated calcium hydroxide solution to undergo curing for 28 days. After curing, the specimens are retrieved and deemed ready for testing.

### 2.3. Test Conditions

The test conditions are shown in Table 2.

### 2.4. Experimental Method

#### 2.4.1. Thermal–Mechanical Coupling Test for Lining Concrete

**Figure 1:**
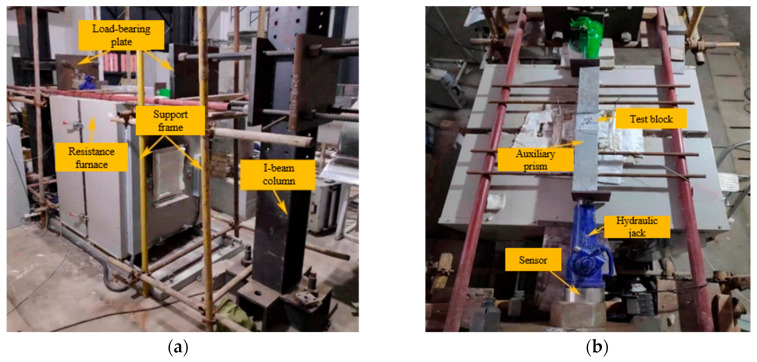
Thermal–mechanical coupling test device. (**a**) Axonometric view; (**b**) Plan view.

The test is intended to simulate the actual process of tunnel lining concrete under fire, which involves heating with constant load using non-uniform and single-sided fire (different from the general concrete fire test). The heating instrument adopts the YFFG768/10QK-3GC (Changsha, China) box-type resistance furnace. The fire is loaded according to the ISO-834 standard heating curve [21], and the fire exposure time is set as 0 h, 0.5 h, 1 h, 2 h. After refitting the resistance furnace, a thermal–mechanical coupling test device is designed, as shown in Figure 1. This test intends to apply a 35% preload to the test block. The test blocks are divided into two groups. One is used for temperature measurement, with pre-embedded SMD thermocouple; the other is used for the thermal–mechanical coupling test.

WA2 (40% coarse aggregate, 1 h fire) was the first block used during the test, and the load ratio was 32%. However, the auxiliary prism was damaged during the cooling process. Thus, in the follow-up test, the load ratio was changed to 28% in order to ensure the smooth progress of the test.

#### 2.4.2. Concrete Static Load Test

The compressive strength of concrete was obtained through the static load test. The test was carried out according to the [20]. The static load test was carried out three times. The first test comprised the 28-day strength test, as well as one test before and one after the thermal–mechanical coupling test, respectively.

#### 2.4.3. CT Scan

The instrument adopted was a phoenix v|tome|x m all-round X-ray micro-focus CT system(Huerth, Germany). A total of 6 blocks were used in the test, which were scanned twice, and the parameters of each scan were the same. The first scan was performed before the thermal–mechanical coupling test to observe the initial defects of the specimen and the distribution of mesoscopic components and to prepare for subsequent numerical simulation by reconstructing the section graph. The second scan was performed to observe the internal damage after the thermal–mechanical coupling test was completed and to compare it with the results of the first scan test to analyze the microscopic deterioration characteristics.

## 3. Results and Discussion

### 3.1. Analysis of Macro Results

#### 3.1.1. Temperature Field

**Figure 2:**
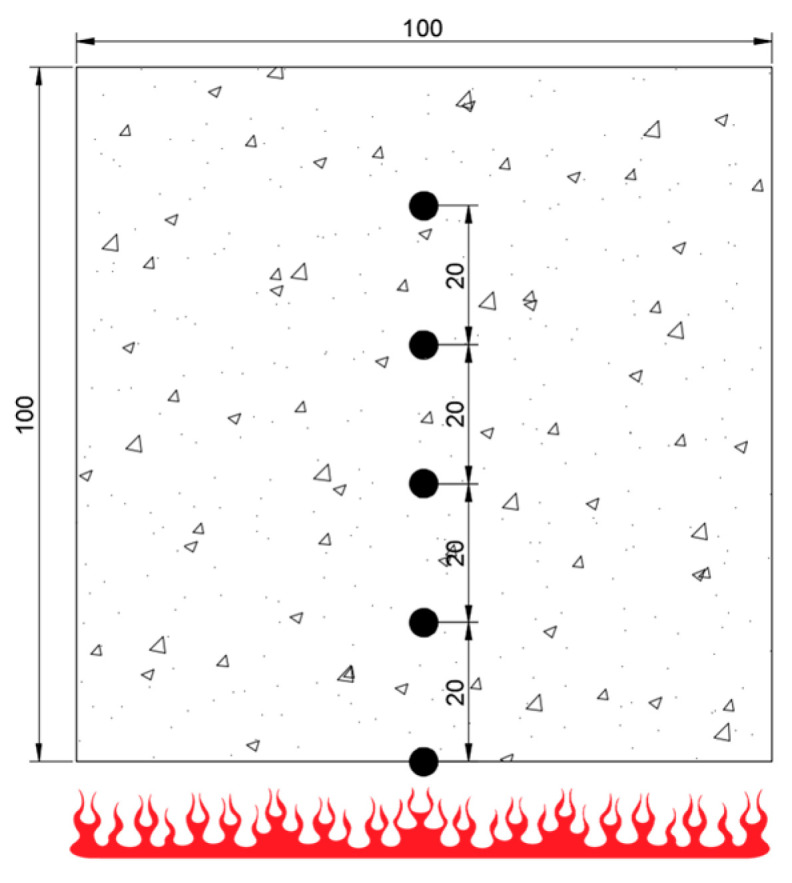
Measuring point layout (unit: mm).

**Figure 3:**
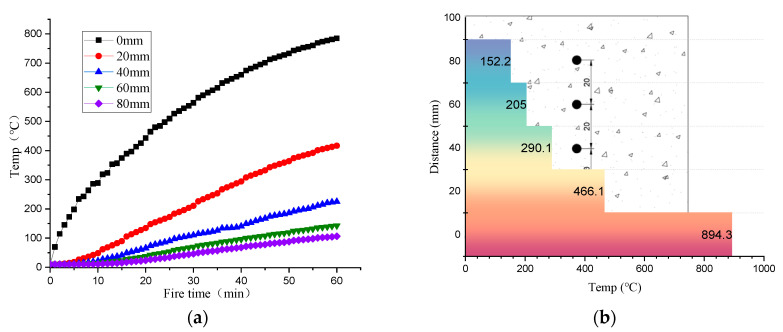
Temperature distribution of concrete with 40% coarse aggregate content. (**a**) Temperature curve at 1 h; (**b**) Temperature distribution at 2 h.

Type K thermocouples with a range of 980 °C and accuracy of ±1.1 °C are built into the test block, and the layout of measuring points is shown in Figure 2. A measuring point is set every 20 mm along the thickness direction of the test block. Taking the concrete with 40% coarse aggregate content as an example (after being exposed to fire), the temperature distribution is shown in Figure 3.

It can be seen that the temperature of each point inside the concrete increases gradually with the increase in exposure time, and higher temperature occurs in the surface layer. However, because the temperature transfer is limited by the material properties, the temperature curve cannot be the same as the heating curve, even on the concrete surface.

**Figure 4:**
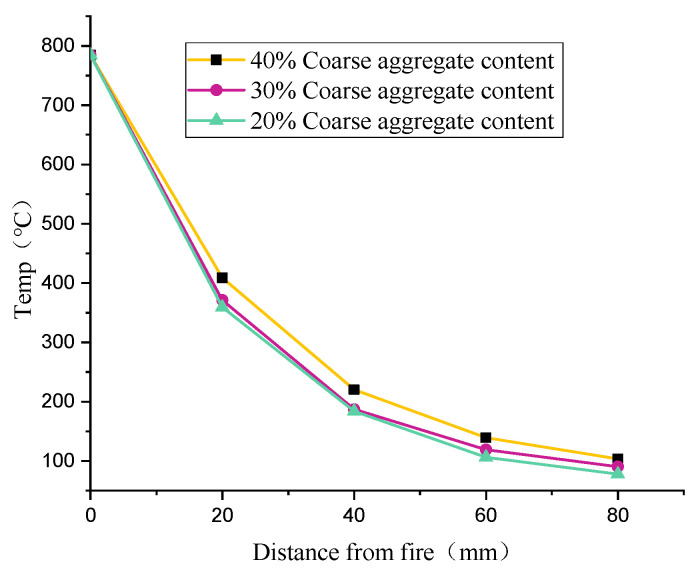
Temperature distribution of concrete with different coarse aggregate contents along the thickness direction.

Figure 4 shows the internal temperature of concrete with different coarse aggregate contents after being exposed to fire for 1 h. Under the same exposure time, the temperature is different. The higher the coarse aggregate content, the faster the temperature transfer to the interior and the higher the temperature at the same position. This is because the coarse aggregate has a high density and low porosity, and the mineral components within are of superior thermal conductivity. In contrast, the mortar composition, which includes water and air, produces poor thermal conductors.

#### 3.1.2. Compressive Strength

**Figure 5:**
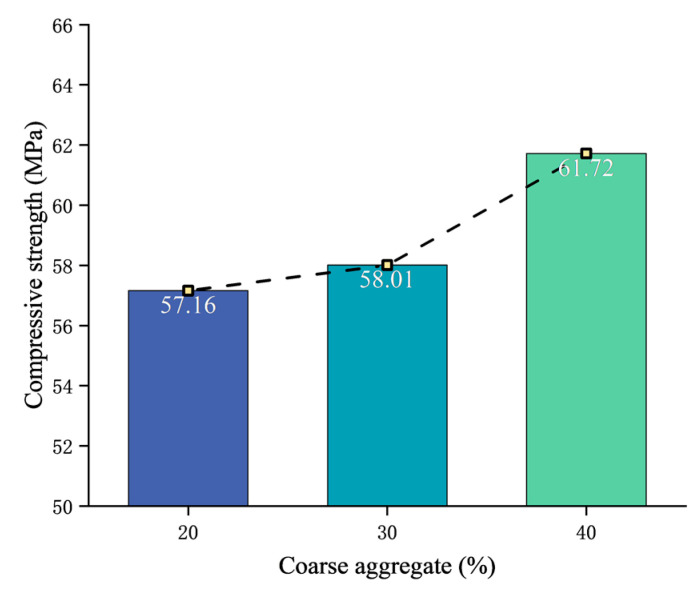
Compressive strength of concrete with different coarse aggregate contents.

**Figure 6:**
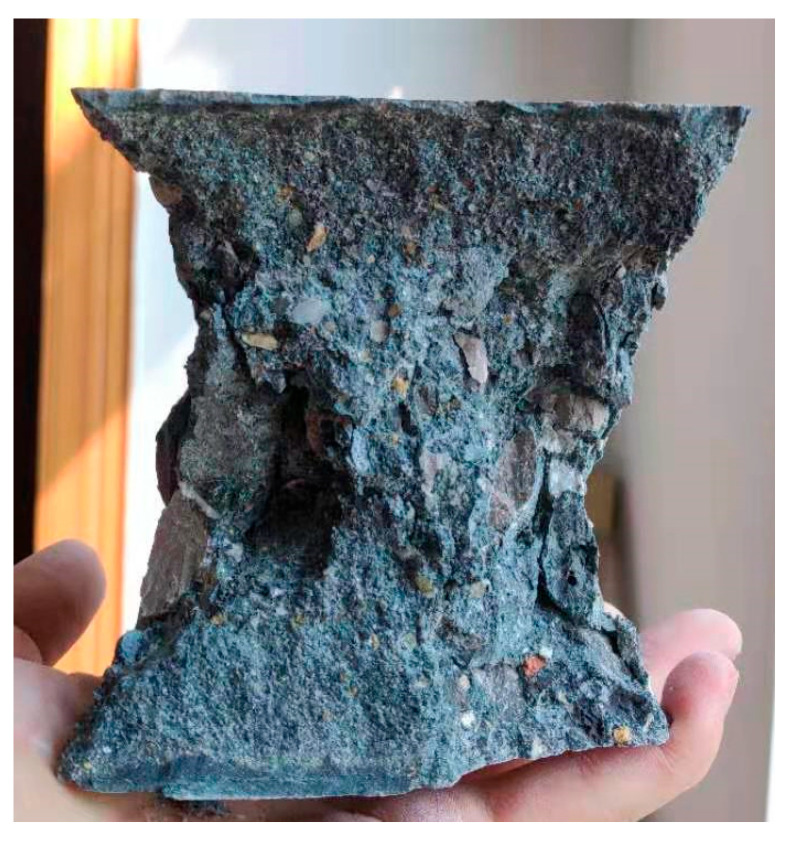
Concrete failure characteristics.

After the test blocks were cured for 28 days according to the standard curing conditions, the compressive strength tests were carried out. The test results are shown in Table 3 and Figure 5. The failure characteristics of the concrete are obvious, showing a wedge-shaped failure, peeling off all around, and a thin waist in the center, as shown in Figure 6.

The strength of the test concrete is greater than the design strength of 50 MPa, which meets the requirements. It can be seen that with the increase in coarse aggregate content, the compressive strength increases gradually. Additionally, the increase in compressive strength when the coarse aggregate content ranges from 30% to 40% is significantly faster than when the content ranges from 20% to 30%. The total aggregate volume fractions of the concrete are 50%, 60%, and 70%, respectively, and the strength variation law found is consistent with the existing research results [22].

The strength changes in different aggregate concretes under different ages were analyzed. The thermal–mechanical coupling test is performed about 90 days after the curing of specimens is completed, and the strengths at 28 days and 90 days are shown in Table 4.

**Figure 7:**
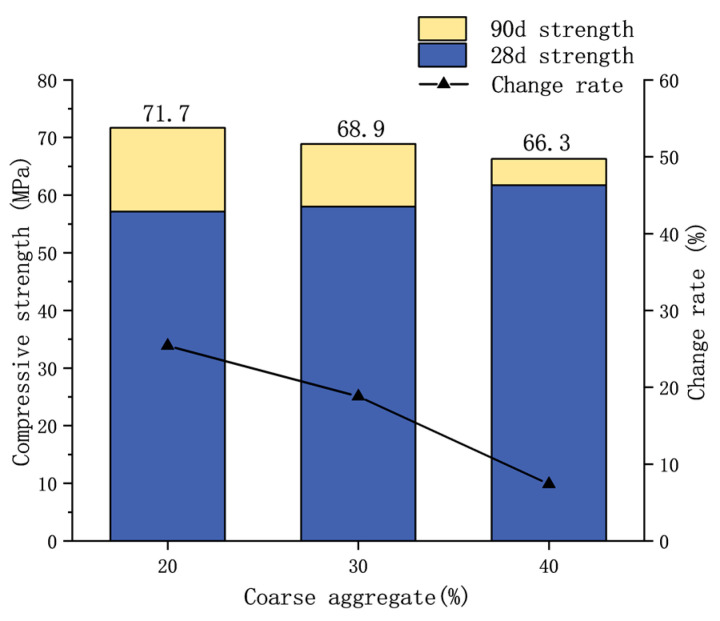
Change rate of concrete strength with different coarse aggregate contents.

It can be seen that the strength of concrete increases over time, and Figure 7 shows that the growth rate of strength decreases with the increase in coarse aggregate content. This is attributed to the fact that concrete with a lower coarse aggregate content—having a higher proportion of mortar—experiences an enhancement in overall strength due to the continuous hardening of the mortar over time. Conversely, concrete with a higher coarse aggregate content tends to exhibit more stable strength in its later stages.

Before and after the thermal–mechanical coupling test, the compressive strength of each test block was measured, as shown in Table 5. The calculation of the strength loss is shown in Equation (1).

(1) $$f=\frac{\sigma_{0}-\sigma_{s}}{\sigma_{0}}$$

In the equation,

*f*—strength loss;

*σ*_0_—initial strength of concrete (here, the strength is measured at 90 days);

*σ_s_*—residual strength after the test.

When *f* = 0, the concrete strength is not lost; when *f* = 1, the concrete strength is completely lost; when *f* < 0, this indicates that the strength is enhanced.

**Figure 8:**
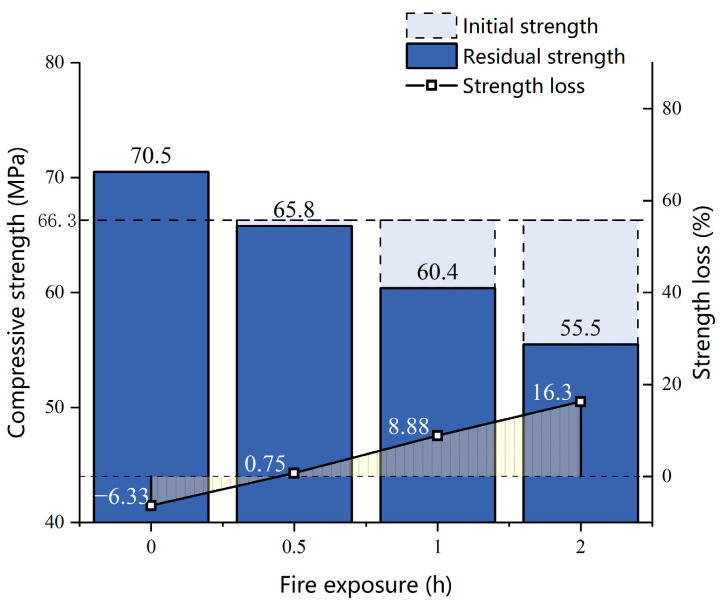
Strength loss of 40% coarse aggregate concrete under different fire exposure times.

Analyzing the strength loss of 40% coarse aggregate concrete (Figure 8), it can be seen that the strength loss is negative when the concrete is subjected to 28% preload without fire, indicating an increase in concrete strength. In contrast, the other test blocks exhibit strength loss under the combined effects of preload and fire exposure, with the strength loss becoming more pronounced as the duration of fire exposure increases.

**Figure 9:**
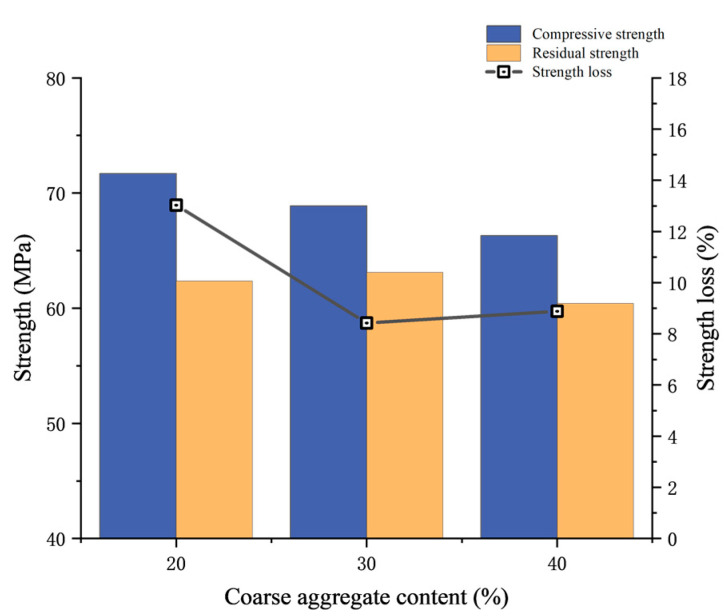
Strength loss of different coarse aggregate concretes under 1 h fire exposure time.

The strength loss of different coarse aggregate concretes after 1 h fire exposure time is shown in Figure 9. The strength loss of concrete with 20% coarse aggregate is the largest, at 13.03%. When the coarse aggregate content ranges between 20% and 30%, the strength loss shows a decreasing trend. However, there is a slight increase at 40%, which is probably due to the difference in preload. It is speculated that under the same load ratio, the strength loss tends to decrease with the increase in coarse aggregate content. This observation suggests that while concrete with a reduced coarse aggregate content demonstrates comparatively higher compressive strength in its advanced age, it concurrently exhibits diminished fire resistance and reduced durability. Such characteristics are disadvantageous for ensuring the safe and sustainable operation of tunnels.

### 3.2. Analysis of Mesoscopic Deterioration Characteristics

#### 3.2.1. CT Image Analysis of Lining Concrete at Room Temperature

**Figure 10:**
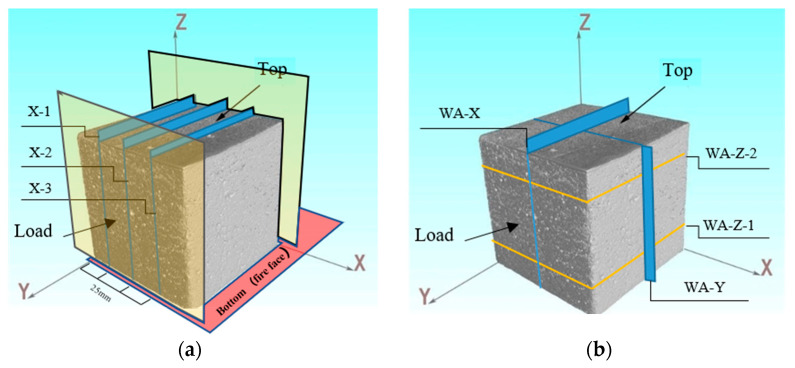
Three-dimensional reconstruction of concrete and the representative layers selection. (**a**) Layers selected at room temperature; (**b**) Layers selected after the coupling test.

The scanned specimens are 100 mm cubes. After scanning, the computer system in the CT is used to reconstruct the image, and the result is shown in Figure 10. When performing two-dimensional image analysis, cross-sectional views in different directions are selected for analysis. In this test, the fire-receiving direction is the *Z*-axis; the fire-receiving surface is the bottom. The top is the free surface when the test block is poured. The loading direction is along the *Y*-axis, and the *X*-axis is unconstrained. When analyzing the specimens at room temperature, only the longitudinal section of the tunnel is selected—that is, the Y-Z diagram.

**Figure 11:**
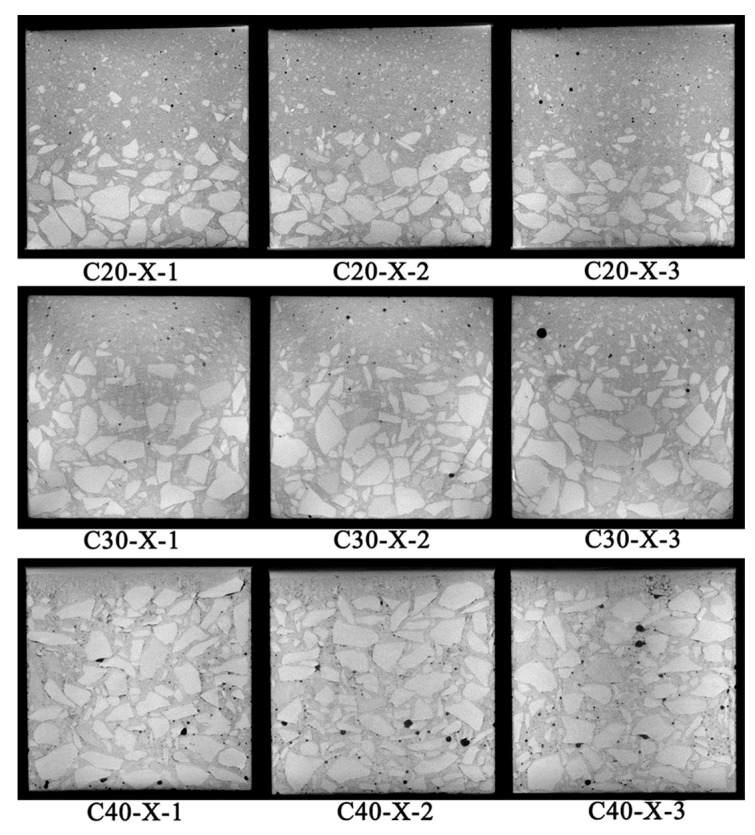
CT scans at room temperature.

The gray value of each pixel in the CT image reflects the density of different materials. The greater the density, the brighter the image, and vice versa. The density of mesoscopic components, such as the pores, mortar, and aggregate, increases in turn; therefore, the aggregate is the brightest in the CT image, presenting an off-white color. The density of the pores is the smallest, appearing black in the picture. The density of the mortar is between that of the aggregate and the pores, and it appears dark gray in the image. The CT image is shown in Figure 11.

The representative layer of room temperature CT scanning is selected every 25 mm (Figure 10a). WA is selected as representative of 40% coarse aggregate content. C20-X-1 represents the view of the first layer, along the X direction, with 20% coarse aggregate content. The remaining labels have similar meanings.

The black part in the figure represents the void. It can be seen that all concretes contain the original pores. The pores are different in shape and size and are mostly distributed near the aggregate, forming a porous area. The appearance of a porous area is due to many factors, such as the side wall effect in the early stage, spontaneous shrinkage in the hardening stage, and the difference in expansion coefficient between the aggregate and the matrix. It can be found that the initial defects of concrete are mainly pores rather than microscopic cracks.

Two phenomena are clearly evident in the CT images. The first phenomenon is the distribution of pores. In concrete with 20% and 30% coarse aggregate, most pores are distributed within the mortar matrix, with few appearing at the bottom. However, in the 40% coarse aggregate concrete, the pores are characterized by a wider distribution and larger diameter, being predominantly located at the bottom of the specimen. The reason for this is that bubbles cannot traverse the coarse aggregate to reach the top during the vibration process. Conversely, in concrete with lower aggregate content, the increased presence of mortar in this region facilitates the upward movement of bubbles. The second phenomenon is the distribution of the aggregates. It can be seen in the Y-Z section that the coarse aggregates are different in the vertical distribution area, showing an obvious vertical accumulation effect [23]; that is, under the action of gravity, most aggregates are distributed at the bottom. There is less aggregate content the closer one gets to the top.

#### 3.2.2. CT Image Analysis of Lining Concrete under Different Fire Exposure Times

In the test, the fire-receiving direction is along the *Z*-axis; the fire surface is the bottom. The loading direction is along the *Y*-axis, and the *X*-axis is not constrained. Taking WA as an example, three directions are selected for image analysis. As shown in Figure 10b, two representative layers are selected along the Z direction, which are 3 mm from the bottom and the top, respectively, and they are recorded as WA-Z-1 and WA-Z-2. Then, select the middle layers along the *X*- and *Y*-axes as representative layers, denoted as WA-X, WA-Y. Select two states for each representative layer, before and after the fire experiment.

**Figure 12:**
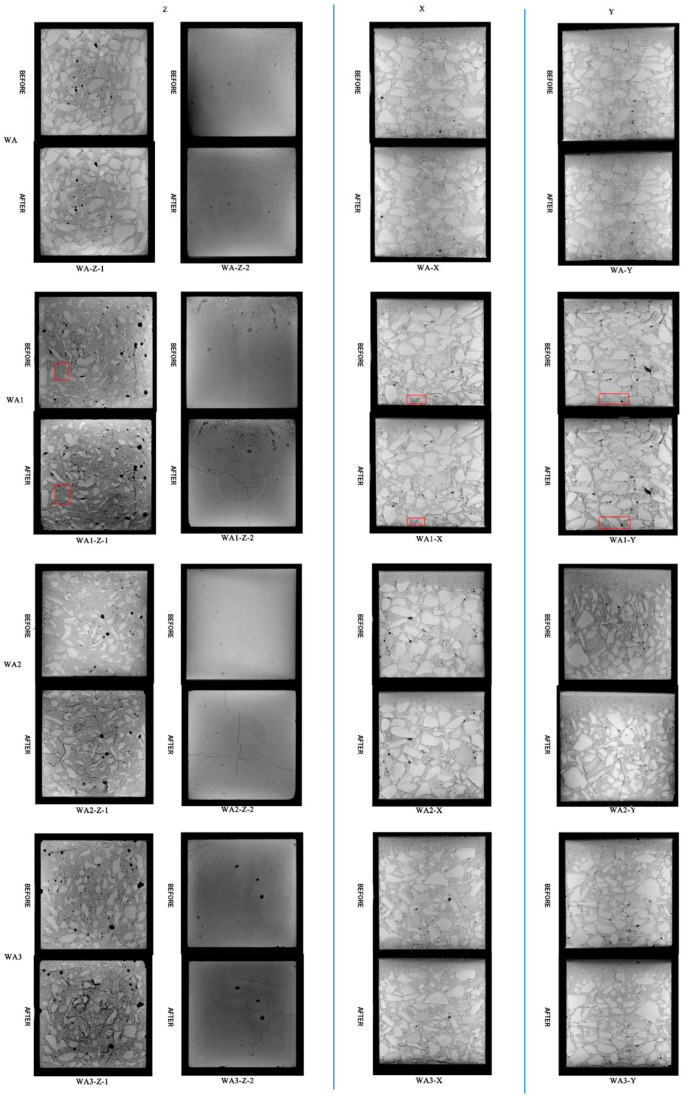
CT images of 40% coarse aggregate concrete under different fire exposure times.

Based on damage to the 40% coarse aggregate concrete under different fire exposure times (Figure 12), the following results can be obtained.

(1) Upon exposure to fire for 0 h and under 28% uniaxial strength, it can be observed that there are no cracks in the concrete, which is consistent with previous research results [24].
(2) There are similarities and differences in damage to the three concrete specimens under different fire exposure times.

The characteristics of mesoscopic deterioration under fire exposure are similar. ① Cracks originate in the interface layer, pores, and mortar. In severely damaged sections, cracks expand and overlap each other after initiation, forming long cracks or piercing cracks. ② The proportion of cracks distributed in the aggregate interface layer is relatively high, followed by that in the periphery of the pores and in the mortar. The number of cracks passing through the aggregate is the lowest. The previous observation indicates that there is a dense distribution area of pores around the aggregate. The phenomenon of cracks being mainly located in the interface layer is closely related to the distribution of pores. Therefore, the interface layer and pores are the main weaknesses of concrete. ③ Concrete with 40% coarse aggregate has regional damage characteristics in the experiment, that is, damages are mainly located in the high-temperature area at the bottom (above 400 °C), the middle area (around 200 °C), and the top of the block. The deterioration in the high-temperature bottom area is relatively the most serious; the damage in the middle area is scattered; cracks appear on the top of each block, but the damage is the lightest. It should be noted that the divisions are mainly based on damage characteristics, and there are still cracks in other areas. ④ The phenomenon of “prominent aggregate expansion” exists in each block; that is, the aggregate expands when heated, and the edge of the aggregate is more obvious and visible in the CT image, as shown in the red area of WA1. This phenomenon is defined as “aggregate protrusion” in this paper.

Differences: ① The range of the bottom damage area is different. The damage areas end at 5 mm, 15 mm, and 25 mm from the fire surface for exposure times of 0.5 h, 1 h, and 2 h, respectively. The longer the fire exposure time, the greater the degree and depth of damage. ② Aggregate protrusion occurs in different areas. The protruding areas are located 10 mm, 15 mm, and 30 mm from the bottom, respectively, for exposure times of 0.5 h, 1 h, and 2 h, where the temperature is basically above 300 °C. Through the scanning electron microscope (SEM), it is found that the flocculent gels in the mortar dehydrate obviously at above 300 °C [25]. At this time, the aggregate cannot be completely wrapped by the slurry, and the aggregate is exposed. The higher the temperature, the more severe the decomposition of calcium silicate hydrate gel and flaky Ca(OH)_2_, which makes exposure of the aggregate obvious. In addition, the volume of limestone increases obviously after 200 °C [26]. Under the joint coupling of aggregate expansion and mortar shrinkage, cracks at the edge of the aggregate are initiated. Finally, the boundary becomes clear. ③ There is a significant difference in damage as the duration of fire exposure varies. For the exposure time of 2 h, cracks form in the bottom fire area and they are wide, and the mesoscopic materials are close to falling off. For the WA2 (1 h, 32% load ratio), a vertical discontinuous micro-crack appears along the loading direction (Y-axis), but it does not appear in the WA3 (2 h, 28% load ratio). This might be attributed to the uneven heating of the concrete, wherein the side facing the fire experiences a more rapid temperature increase, leading to more significant expansion. Conversely, the side away from the fire—due to less heat exposure—expands less, ultimately causing the concrete to bend toward the fire, putting tension on the bottom. The application of a considerable uniaxial load also leads to the expansion of the concrete toward the unconstrained horizontal direction, further intensifying the tensile stress at the bottom. Eventually, the concrete undergoes tensile cracking from the bottom, developing cracks, which propagate inward. This suggests that uniaxial load is the primary factor in crack formation rather than temperature. However, temperature also plays a role in crack development: in WA3, for example, the crack extends from the bottom to the surface. It can be inferred that while the load tends to cause comprehensive structural damage, the temperature predominantly leads to regional damage—usually due to inconsistencies in expansion under uneven heating.

#### 3.2.3. CT Image Analysis under Different Coarse Aggregate Contents

**Figure 13:**
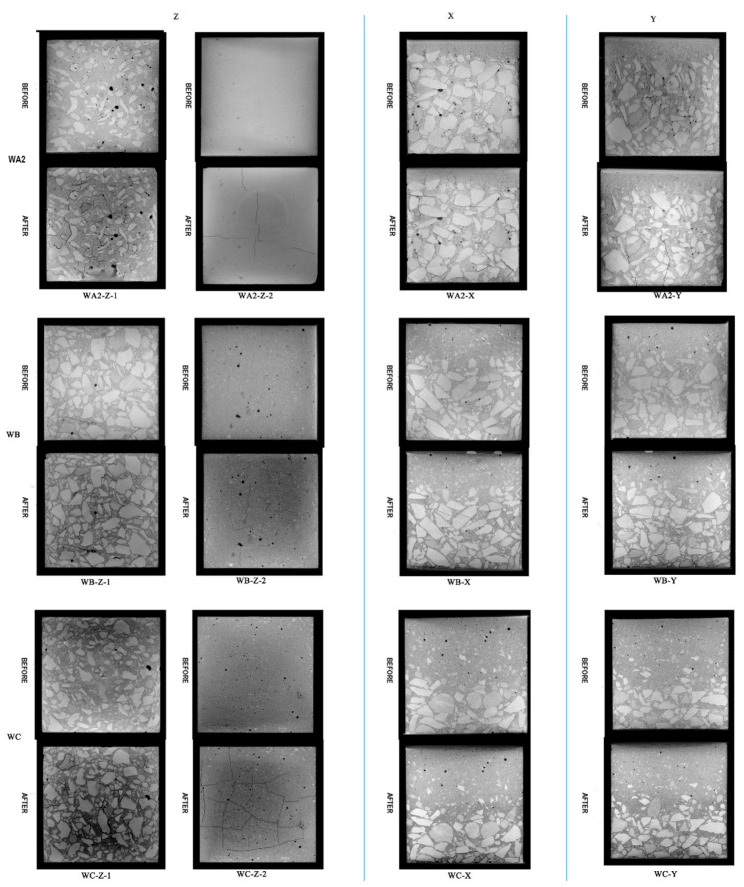
CT images of different coarse aggregate concretes under 1 h fire exposure.

Through the comparison of concrete with different coarse aggregate contents under the same fire exposure time (Figure 13), the following conclusions can be drawn.

① In 20% coarse aggregate concrete, cracks are mainly located in the interface layer and mortar. Lower aggregate content leads to a more significant degradation in the mortar and a higher incidence of cracks. This is because lower aggregate content results in a higher proportion of mortar, which is less resistant to thermal and mechanical stresses than aggregates.
② In concrete with 20%, 30%, and 40% coarse aggregate contents, the thicknesses of severely deteriorated areas at the bottom are 25 mm, 20 mm, and 15 mm, respectively. The damage is more pronounced at the bottom of the concrete samples, with lower aggregate contents showing deeper damage. This is because aggregates can distribute and possibly reduce stress within the mortar matrix, mitigating the extent of thermal and mechanical damage.
③ The 20% aggregate content concrete shows degradation mainly at the bottom, where the temperature is high, and in low-temperature areas on the opposite side, where aggregates are absent, which could not dissipate the heat and stress. In contrast, higher aggregate contents (30% and 40%) show a more distributed degradation, including in the middle areas, likely due to the aggregates’ role in transferring heat and stress more evenly throughout the sample.

### 3.3. Correlation Research on Macro and Micro Damage of Lining Concrete

#### 3.3.1. Definition of the Damage Variable

In the above analysis, it can be seen that concrete exhibits an internal deterioration phenomenon under the thermal–mechanical coupling effect. An appropriate damage variable is required to quantitatively evaluate the damage. Over the years, many studies have defined a variety of damage variables through theoretical and experimental results [27,28,29,30,31,32,33,34]. However, most of those damage variables can only indicate mesoscopic damage by obtaining macroscopic mechanical properties. This method is indirect, and it cannot reflect the essential characteristics of mesoscopic damages.

According to the previous content, cracks are the main feature of concrete mesoscopic deterioration, and cracks are initiated in the pores. Therefore, a quantitative analysis of cracks or porosity can reflect mesoscopic damage characteristics. In this paper, an image statistical software (VGSTUDIO MAX 3.3) is used to quantitatively study the overall 3D distribution of cracks in various states of concrete. Referring to Refs [33,34], the change in crack ratio is used to define a damage variable “*D*”, which reflects the mesoscopic deterioration of concrete, as shown in Equation (2):

(2) $$D=1-\frac{1-\rho_{n}}{1-\rho_{0}}=\frac{\rho_{n}-\rho_{0}}{1-\rho_{0}}$$

In the equation,

$\rho_{n}$—crack ratio of concrete in a certain state;

$\rho_{0}$—crack ratio of concrete at the initial state.

Define the prepared specimen as being in a non-destructive state when *D* = 0; when the specimen is completely crushed, *D* = 1; when *D* < 0, this indicates that the crack rate is reduced, and the specimen is strengthened. Therefore, as long as the crack ratio is obtained, the mesoscopic damage degree can be quantified by the equation.

The crack ratio and damage variable of each specimen before and after the test are shown in Table 6.

In the table, there is little difference in the crack ratio of concrete with 40% coarse aggregate before the test. For comparison with other concretes with different coarse aggregate contents, the average crack ratio of 0.65 is used for 40% coarse aggregate concrete.

**Figure 14:**
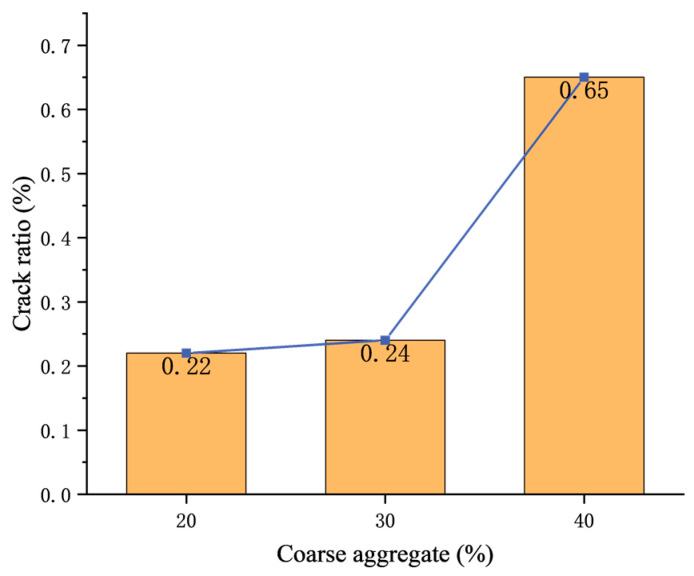
Relation between crack ratio and coarse aggregate content.

Figure 14 shows the relation between the crack ratio and coarse aggregate content before the test. It can be seen that the greater the coarse aggregate content, the higher the crack ratio.

**Figure 15:**
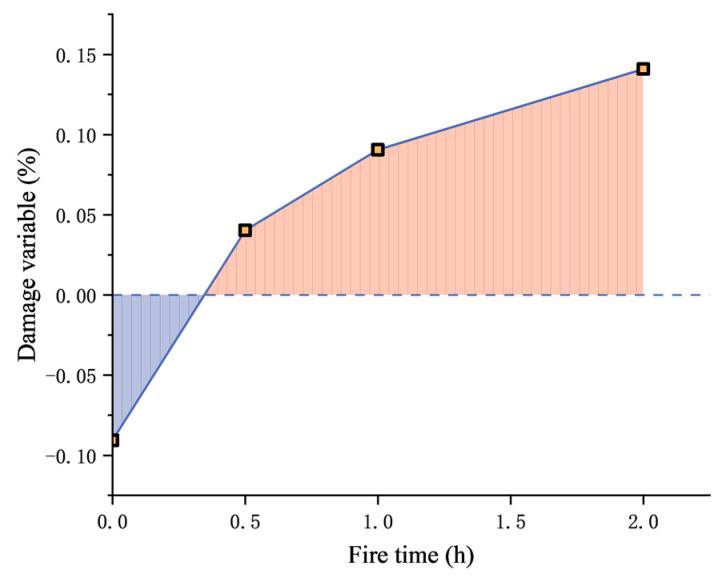
Relation between D and fire exposure time (40% coarse aggregate concrete).

Figure 15 shows the relation between *D* and fire exposure time of 40% coarse aggregate concrete. It can be seen that mesoscopic damage is a process, which increases with fire exposure time. Under constant load and no fire condition, the damage variable is negative. This is because the micro-cracks are compacted under the load, resulting in a decrease in the crack ratio. When subjected to fire for 0.5 h, *D* is positive, but the value is small, indicating that the concrete sustained new mesoscopic damage under fire. The damage degree is low, which offsets the decrease in compaction. Afterward, the damage variable increases with the expansion of fire exposure time.

**Figure 16:**
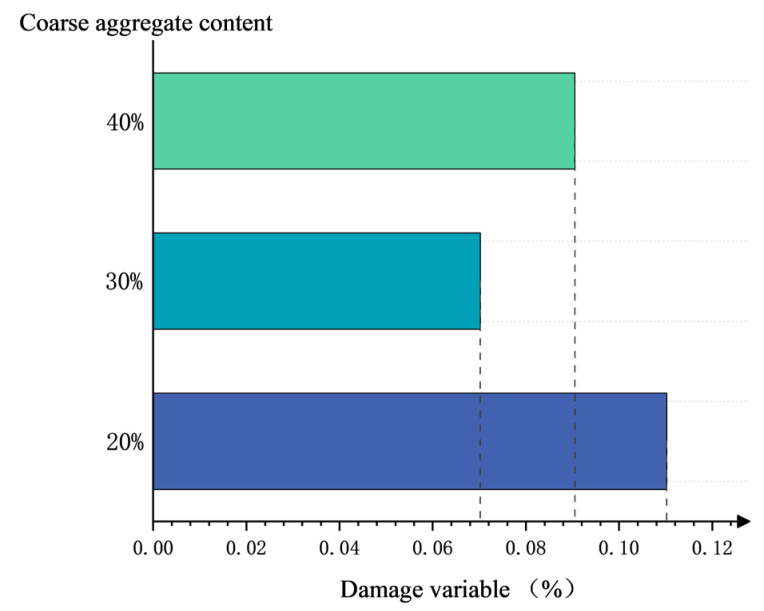
Change in damage variable with the content of coarse aggregate (1 h fire exposure).

Figure 16 shows the change in the damage variable with the content of coarse aggregate under 1 h fire exposure. The damage variable decreases with the increase in coarse aggregate content under the same fire exposure time, indicating that the higher the coarse aggregate content, the stronger the fire resistance of concrete. However, *D* increases in 40% coarse aggregate concrete, which is probably due to the relatively large load ratio (32%; the others are 28%). Vertical cracks form at this load, which increases the damage.

#### 3.3.2. Correlation Analysis

**Figure 17:**
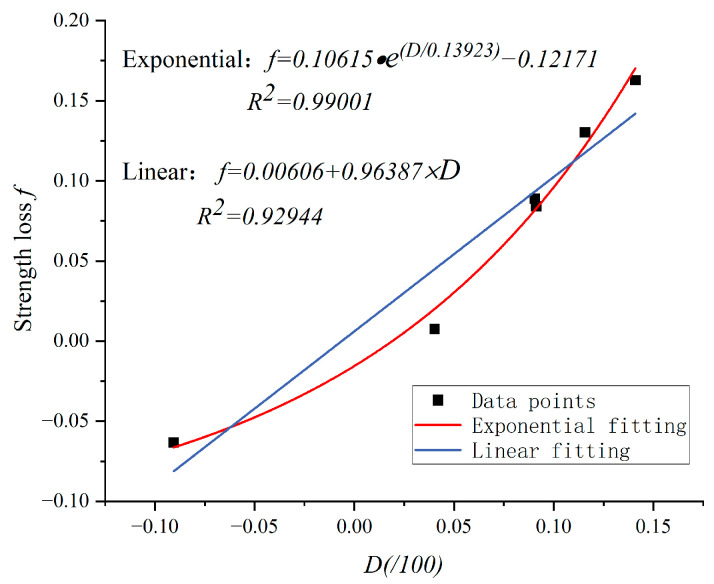
Relationship between D and strength loss.

This section studies the relationship between mesoscopic damage variables and macroscopic mechanical properties, mainly considering the loss of compressive strength. According to the distribution of data points, linear and non-linear methods (exponential function) are chosen for fitting. The results are shown in Figure 17. It can be seen that the goodness of fit of the linear function is worse than that of the exponential function, whose R^2^ is 0.99. Therefore, the relationship between strength loss and the damage variable can be described by the exponential function.

It can be seen in Figure 17 that the strength loss increases with the damage variable, and the increasing rate is slow in the early stage but fast later. When the concrete reaches the ultimate strength and is damaged, from a mesoscopic point of view, some aggregates and mortar bodies still maintain good integrity at this time. This is why *D* does not reach 1. Only when the concrete is completely crushed will *D* reach 1. From this point of view, strength loss and the mesoscopic damage variable do not have a linear correlation; therefore, the exponential function can better describe the actual relation.

The significance of this function lies in measuring changes in the porosity of concrete before and after a fire to estimate its load-bearing capacity and assess the damage levels and safety. This method is simpler and more convenient than conducting load-bearing tests on concrete components. However, further research into the relationship between meso damage variables and strength loss is necessary to establish a more accurate correlation between the two.

## 4. Conclusions

In this paper, a thermal–mechanical coupling test device was modified to simulate the state of concrete under real tunnel fire. Combined with CT technology, the macroscopic properties and microscopic characteristics of concrete were observed, and the relationship between them was explored. The specific conclusions are as follows.

1. Under the same thermal–mechanical coupling condition, the lower the content of concrete coarse aggregate, the weaker the fire resistance;
2. Under the same fire exposure time, the higher the content of concrete coarse aggregate, the faster the temperature transfer to the interior. This is due to the difference in mortar content, whose thermal conductivity is worse than that of the aggregate.
3. Mesoscopic deterioration characteristics of different coarse aggregate contents and fire exposure times were observed, mainly reflected in mesoscopic damage phenomena, such as pores’ distribution, regional damage characteristics, the evolution of cracks, and the “aggregate protrusion” phenomenon.
4. Based on the crack ratio, the microscopic damage variable D is defined to quantify microscopic damage. The longer the fire exposure time, the greater the damage variable; under the same fire exposure time, the damage variable shows a descending trend with the increase in coarse aggregate content. Finally, the relationship between mesoscopic damage variable and the loss of compressive strength is established, which can be fitted with the exponential function. The change in macroscopic mechanical properties can be predicted by this function from the perspective of mesoscopic damage.

However, in this paper, the applied load was relatively small—only 28% of the ultimate load—and the research failed to study the damage law under different load conditions. Moreover, the lining is subjected to eccentric force in the real world; the uniaxial force used in the test could not reflect a real loading situation. In addition, the change in coarse aggregate content was the only factor considered for the mesoscopic components, and the change range of aggregate content was not sufficiently accurate. These issues need to be reconsidered in future research.

## Figures and Tables

**Table 1:** Concrete specimen mix (kg/m^3^).

Coarse Aggregate	Water–Binder Ratio	Water	Cement	Fly Ash	Slag	Big Gravel	Small Gravel	River Sand	Superplasticizer
40%	0.35	147	189	105	126	733	314	775	4.2
30%	0.35	228	292	163	195	563	242	606	0.4
20%	0.35	318	408	227	272	375	161	404	0.16

**Table 2:** Test conditions.

Purpose	Test Type	Fire Exposure Time/h	Load Ratio ^1^	Coarse Aggregate Volume Fraction/%	Specimen Number	Total Number ^2^
Mesoscopic observation	CT scan (before and after fire test)	0	None	40	WA (WAT)	12 (including 6 temperature test specimens)
		0.5	Yes	40	WA1 (WAT1)	
		1			WA2 (WAT2)	
		2			WA3 (WAT3)	
		1		30	WB (WBT)	
				20	WC (WCT)	
Strength calibration	Static load test	/	None	40	WS1–WS3 WS1′–WS3′	18 (WS1′–WS9′ are used to obtain strength parameters before fire test)
				30	WS4–WS6, WS4′–WS6′	
				20	WS7–WS9, WS7′–WS9′	
Influence of fire and mesoscopic components	Fire test	0.5	Yes	40	WA1	5
		1			WA2	
		2			WA3	
		1		30	WB	
				20	WC	
Mechanical performance test after fire	Static load test	0	Yes	40	WA	6
		0.5			WA1	
		1			WA2	
		2			WA3	
		1		30	WB	
				20	WC	

^1^ The load ratio is defined as the quotient of the load applied to the specimen during the test and the specimen’s ultimate compressive strength. ^2^ Apart from one set of specimens designated for strength calibration, the remaining specimens were cast in two identical sets: one for scanning purposes and the other for temperature measurement (labeled in formats such as WAT). The total number of specimens is 30.

**Table 3:** Compressive strength at 28 days.

Coarse Aggregate Content/%	Compressive Strength/MPa
20	57.16
30	58.01
40	61.72

**Table 4:** Concrete strengths under different ages.

Type	28-Day Strength/MPa	90-Day Strength/MPa	Rate of Change/%
40% coarse aggregate	61.72	66.3	7.4
30% coarse aggregate	58.01	68.9	18.8
20% coarse aggregate	57.16	71.7	25.4

**Table 5:** Concrete compressive strength before and after the coupling test.

Name	Compressive Strength *σ*_0_/MPa	Residual Strength *σ_s_*/MPa	Strength Loss *f*/%
WA	66.3	70.5	−6.33
WA1	66.3	65.8	0.75
WA2	66.3	60.41	8.88
WA3	66.3	55.5	16.3
WB	68.9	63.1	8.42
WC	71.7	62.36	13.03

**Table 6:** Concrete crack ratio and damage variable.

Name	Crack Ratio before Test/%	Crack Ratio after Test/%	Damage Variable/%
WA	0.67	0.58	−0.0906
WA1	0.64	0.68	0.0403
WA2	0.63	0.72	0.0905
WA3	0.67	0.81	0.1409
WB	0.24	0.31	0.0702
WC	0.22	0.33	0.1102

## Data Availability

The data presented in this study are available on request from the corresponding author.
